# A critical reflection on the technological development of deep brain stimulation (DBS)

**DOI:** 10.3389/fnhum.2014.00730

**Published:** 2014-09-17

**Authors:** Christian Ineichen, Walter Glannon, Yasin Temel, Christian R. Baumann, Oguzkan Sürücü

**Affiliations:** ^1^Institute of Biomedical Ethics, University of ZurichZurich, Switzerland; ^2^Department of Philosophy, University of CalgaryCalgary, CGY, Canada; ^3^Department of Neurosurgery, Maastricht University Medical CenterMaastricht, Netherlands; ^4^Department of Neurology, University Hospital ZurichZurich, Switzerland; ^5^Division of Neurosurgery, University Hospital ZurichZurich, Switzerland

**Keywords:** deep brain stimulation, technology, development, innovation, functional neurosurgery, stereotactic operation, ethics

## Abstract

Since the translational research findings of Benabid and colleagues which partly led to their seminal paper regarding the treatment of mainly tremor-dominant Parkinson patients through thalamic high-frequency-stimulation (HFS) in 1987, we still struggle with identifying a satisfactory mechanistic explanation of the underlying principles of deep brain stimulation (DBS). Furthermore, the technological advance of DBS devices (electrodes and implantable pulse generators, IPG’s) has shown a distinct lack of dynamic progression. In light of this we argue that it is time to leave the paleolithic age and enter hellenistic times: the device-manufacturing industry and the medical community together should put more emphasis on advancing the technology rather than resting on their laurels.

## Introduction

Early lesion studies in humans and translational preclinical research using laboratory models as well as concomitant early stimulation experiments, pharmacological treatment approaches with L-DOPA in 1968 and the identification of circuit physiology in the early 1980’s have led to the development of neuromodulation techniques such as deep brain stimulation (DBS). In contrast to medication-based approaches, knowledge gained from DBS as both a probe and modulator of the underlying neural circuitry resulted in a new way of describing and understanding (neuro)-pathologies (top-down approach). Early stimulation experiments such as those using non-human primates (Kringelbach et al., 2010) were a glowing example of such development. However, we still struggle with explaining the mechanisms of action of DBS as Montgomery stated in his paper on logical pitfalls on DBS results and mechanisms of action (Montgomery, 2012). 2012 marked the 25th anniversary of modern DBS. Although DBS is being performed increasingly in centers worldwide, not much has changed regarding the integration of new technological know-how. This poses the distressing question of whether there is a duty to overcome this lack of progress. The general picture of an apparent arrested development does not at all light up when analyzing the approximately 40 years of emerging neurostimulation technologies which were adapted into therapies by neurosurgeons for different conditions during the 1970s (Gardner, 2013), besides early stimulation experiments on patients e.g., by Robert Heath and Jose Delgado. Compared to other technological advances in different domains ranging from consumer electronics to medical applications, the technological advance of DBS devices seems to be almost nonexistent.

Furthermore, DBS is at a critical turning point which is characterized by a subsequent collapse of the “last-resort” connotation DBS once had as a treatment for refractory disorders. Indeed, beyond broadening indications to younger patients with well-studied diseases, even questionable indications arise (Hariz, 2012). Is it legitimate to use the same electrodes and stimulation techniques in every region of the brain for movement or psychiatric disorders? And isn’t it plausible that local anatomy may need different hardware/software combinations? Will old concepts of lesioning with new technologies, e.g., high-intensity focused ultrasound, replace DBS in some domains (Lipsman et al., 2013)? Is it possible that genetic and cell culture technologies could overrun the “gold standard” DBS for Parkinson’s disease in the future (Lindvall, 2013)?

## Technological developments: different contexts—same patterns?

**Figure 1:**
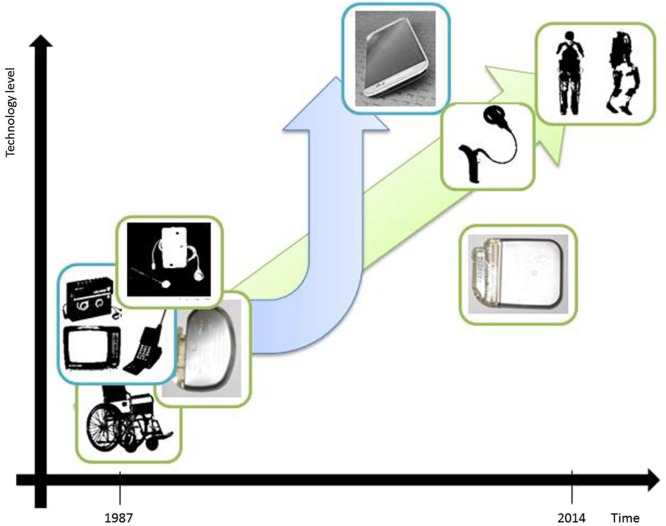
**Illustrative examples of technological developments in the past 25 years which refer to miniaturization aspects and ground-breaking change of technological complexity**. Wheel chair bound patients could in the meanwhile benefit from the possibility of full mobilization due to an exoskeleton device (adapted from: http://www.medicalspro.com/manual_wheelchair.php, http://www.designboom.com/technology/elegs-exoskeleton-by-berkeley-bionics/). External devices of cochlear implants have become remarkably smaller (adapted from: http://www.enttoday.org/details/article/4550891/History_of_the_Cochlear_Implant.html, http://www.audiology.org.nz/Userfiles/Image/implant6_lge.jpg). Television, Walkman and Mobile Phone have become a single, extremely sophisticated multifunctional tool with wireless connections to other technical devices and integrating Internet (Smart-Phone). (adapted from: http://www.radiomuseum.org/r/waltham2_tele_star_4004.html, http://www.telegraph.co.uk/technology/5733286/Sony-Walkman-in-pictures.html?image=4, http://www.webdesignerdepot.com/2009/05/the-evolution-of-cell-phone-design-between-1983-2009/; IPG & mobile phone images used by courtesy of owner).

Figure 1 depicts some of the technological advances that have had applications both inside and outside the medical domain. For our purpose it is sufficient to state that the driving factors for technological evolution and innovation are the following:

- reduction of size dimensions
- increase of complexity and variability of tasks which can be executed

Lewis H. Morgan’s stage model of social evolution involves the analysis of technological milestones and declares technological progress to be the primary factor driving the development of human civilization. On the basis of this model, there is the question of how to interpret our current evolutionary stage by focusing on DBS device advances (see Figure 1). Have we progressed beyond Morgan’s savagery stage?

Pragmatically we think it is fair to say that in the context of DBS, neither (1) nor (2) have yet been satisfied (also see Figure 1 for a direct, schematized visual comparison). The possibility of spatial steering brain stimulation (Martens et al., 2011; Hariz, 2014) or adaptive DBS (Little et al., 2014) barely rises at the horizon. Furthermore, settings are pre-determined per electrode site and can be changed only in a very restricted way due to the missing feedback path (Eberle et al., 2011). Also it appears as if technological trends from neighboring disciplines (i.e., “technology-transfer” (Morlacchi and Nelson, 2011)), such as the gravitation-field sensor system in the context of spinal cord stimulation or nano-technology have not in the least been adopted by the DBS industry. Currently, greater emphasis is put on the issue of MRI safety of DBS implants due to diagnostic highfield-MRI (Paek et al., 2013), which is increasingly being used in clinical work.

## A thought-provoking piece of surgical evidence

**Figure 2:**
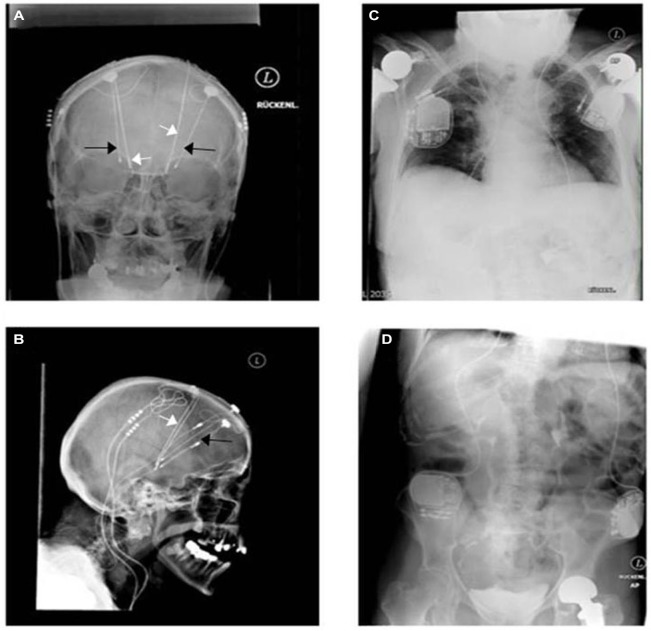
**A unique witness of DBS history**. Bilateral monopolar electrodes were implanted within the internal globus pallidus (GPi) in 1998 in a patient having suffered from typical Parkinson’s disease since 1984. Predominant disabling dyskinesia improved significantly afterwards (black arrows: more lateral in **(A)** and more anterior in **(B)**). Because of re-emerging motor fluctuations without further optimization possibilities of the monopolar GPi electrodes (probably side effects), bilateral quadripolar electrodes (white arrows) were then implanted within the STN in 2001 accounting finally for four intracerebral electrodes and four pulse generators **(C,D)**. The patient profited for around 3 years before developing late stage symptoms, like frequent falls, voice alteration, On-dystonia in her lower extremities and cognitive decline. The two GPi generators were explanted in 2009 as additional beneficial effects were not seen.

Results of randomized controlled trials have just recently been published without major differences regarding outcome of STN-DBS vs. GPi-DBS (Weaver et al., 2012; Odekerken et al., 2013). Figure 2 depicts the desperate search and use of the newest technology available for treating a patient with Parkinson’s disease. Both physician and patient were willing to try the best treatment possible for alleviating the symptoms of this disabling disease. The X-ray images were acquired in 2009 before exchange of the implantable pulse generators (IPGs) connected to the STN (subthalamic nucleus) electrodes. One could speculate that a change to a more sophisticated technological system in GPi (globus pallidus internus) stimulation could have served the purpose of inducing a therapeutic effect without undergoing additive stereotactic surgery. Those images unmask the hopeful will to change treatment in regard to hardware and target without having understood the failure of the monopolar GPi stimulation in this patient. The transition from mono- to quadripolar electrodes still remains the maximum of technological progress today. In daily clinical work, there is a subliminal frustration on the imbalance of having more than 10,000 programming possibilities, e.g., frequency, pulse width and amplitude, without being able to optimize stimulation for the individual patient (Volkmann et al., 2006; Ricchi et al., 2012). Instead, expert recommendations remain the evidential basis of setting parameters. Regarding multiple joint prostheses and four IPG’s, this patient has reached nearly the limit of medical implant costs.

## Technological considerations

The recent advances of increased magnetic field strength observed in MRI-system technology should ultimately lead to an improvement in the accuracy of electrode positioning. This is particularly important given the high interindividual anatomical variability of structures such as the STN, because personalized, image-based targeting provides higher accuracy than atlas-oriented targeting (Ashkan et al., 2007). Moreover, Zaidel et al. (2010) showed optimal clinical efficacy by determining local electrophysiological parameters characterized by increased β-oscillatory activity on multi-unit recordings rather than by pure anatomical analysis.

Today, the impossibility of detecting specific alterations in underlying brain activity depending on the condition being treated is a major limitation. Do we pay tribute to extremely complicated homeostasis patterns of a billion years of evolution, e.g., circadian neuroendocrine interaction and electrochemical networks? Currently, IPGs can be programmed through modifying frequency, pulse width, current output (amplitude), interleaved mode (Baumann et al., 2012) between contacts and limited possibilities of groups/cycling modes. In routine clinical practice, the physician usually takes “standard” programs known from the literature. In case of side effects of medication and stimulation interaction in the variable disease patterns, “trial and error” within thousands of programming possibilities can be frustrating, extremely time-consuming and may require hospitalization of “difficult” patients (Volkmann et al., 2006; Hariz, 2012). Steering the electrical current and granting MRI compatibility are recent and important developments to solve the problem of side effects, energy consumption and harmless hardware-tissue interaction (Hariz, 2014). Given that the brain uses dual-mode communication and feedback strategies, an effective treatment needs to detect and return both real-time monitoring of chemical neurotransmitter release levels as well as electrical firing patterns (Rosin et al., 2011)—in other words, closed loop self-regulatory systems (Grahn et al., 2014). One of these systems is currently under investigation at multiple centers worldwide without any information, yet on future efficacy or availability. The advances of real-time, instantaneous neurochemical and electrophysiological sensing combined with feedback-guided anticipatory adjustment and sophisticated electrode (lead) design (Arcot Desai et al., 2014; Hariz, 2014; Kent and Grill, 2014) could lead to a fully integrated, small and high precision, low-power circuit supporting a wireless neuromonitoring and neuromodulation system (Shah et al., 2010). Hence, avoiding thick, unlabeled cables and clumpy IPGs would reduce acute and long-term morbidity as hardware complications still remain frequent adverse events in DBS surgery in many centers (Hamani and Lozano, 2006).

Additionally, there is a lack of monitoring and integrating disease symptoms together with general health and activity status of the patients. There is no automatic or remote report system, e.g., of “red flags” of symptoms or hardware-related problems, not even for precise battery life duration.

We argue therefore that the time has come to focus on advancing the DBS system in terms of technological properties to better meet individual patient needs, leading to more effective symptom control, improved patient quality of life and reduced healthcare costs.

Given the importance of DBS, the lack of biomarkers for many complex conditions and the associated need for a critical and objective pre- and post-DBS therapy evaluation, we argue that we should emphasize improving means for a holistic evaluation of patient’s clinical status. As Gardner (2013) brought to the fore the decisive role of the invention of a (synthetic) UPDRS rating scale for the emergence of DBS, we also have a duty to critically question whether the creation of such an objective numerical variable in order to have a basis for comparing pre-and post-stimulation effects fully embraces the patient and results in improvement of symptoms. Gardner further states: “Thus while such a tool may generate the necessary “objective evidence” to legitimate the intervention and enable device manufacturers to market their device, it may not be capturing clinical changes that are meaningful to patients and their families” (Gardner, 2013). Thus, quantified rating scale scores have to be complemented by qualitative measures (Bagby et al., 2004).

## Economic considerations

Of course, medical products are under stricter regulatory processes than consumer goods, and doctors or patients are not simple consumers. To the best of our knowledge, one single company held the monopoly for DBS application systems for almost 20 years. Even after the introduction of two other key companies, besides newly emerging vendors highlighted in a recent market study (Global Deep Brain Stimulator Market 2012–2016),^1^ there was no ground-breaking new technological development. Not more than minor changes in programming possibilities, lead and fixation design, and battery properties have occurred. This is problematic since we find ourselves in a context of severe diseases which depend on innovative treatment options. The lack of favorable (see Hashmi, 2013) competition for product-innovation for developing safer and more effective neuromodulation techniques stands in marked contrast to the situation of other technological domains outside the medical field, such as the mobile phone or automobile industry. Here we face a highly dynamic progression of consumer-oriented new developments. In other, more competitive domains within medicine, such as orthopaedic surgery/applications, a more innovative landscape can be observed. A lack of competition may be one reason why we are confronted with a shortage of technological progression in the DBS field. By looking at the economic literature, and by keeping in mind that the health-economic sector is different, one could argue that more product market competition can under certain circumstances increase product-innovation (Aghion et al., 1999, 2009; Aghion and Griffith, 2005; Hashmi, 2013; Roper et al., 2013).

In the DBS context, it is unclear why we do not face a pronounced conflict between attending physicians and industry regarding differing claims. Possibly there is not enough demand (supply-and-demand situation) verbalized by the medical establishment which could explain a lack of innovation potentially due to daily work-intensity and time pressure, regulatory hurdles, investment return considerations, installed base effects, a lack of knowledge-transfer from basic science to users in clinic or a low level of sensibility to grasp the responsibility to strive for more benefit to the patient.

One could potentially identify a subliminal tendency towards investing in other indications, thereby increasing the number of implants instead of improving quality.

## Ethical considerations

We argue that there is a multidimensional obligation to promote innovation which should include all agents involved in the process of helping patients in need. We restrict ourselves to the two domains already highlighted above, namely the medical community and the medical device industry. Those domains should both promote innovation since they have complementary rather than competing goals. In the following, we focus briefly on the medical community on the one hand and more extensively by the use of a framework-based argumentation on the medical device industry on the other.

It appears that the medical establishment has failed to build on the dynamic treatment breakthroughs of the past due to overconfidence and a lack of vision. The initial clinical efficacy in resolving some symptoms obscured the once broadly heralded, noble aspirations of gaining deeper understanding of DBS. Even though the subsequent unraveling of side effects could have driven clinicians to obtain deeper scientific knowledge as well as to optimize treatment, this unfortunately was not pursued with the required vigor.

We believe that it is therefore time to include some ethical reflection in the debate. One could argue that industry and the medical community both have a duty to invest more in technological advances by concomitantly ensuring that treatments are safe and effective (Hofbauer et al., 2013). Generally more emphasis should be put on basic scientific advances (Hamani and Temel, 2012), thereby reaching a level of development which in the end will, as a manifestation of a sensitive pleading for the centrality of patients, help those most in need. Also the question of an ethical responsibility to strive for the best and latest technology for the treatment of one of our most sheltered organs, our brain, should be discussed.

An ethical framework is needed to make this normative claim more transparent and more systematically structured. We believe that interposing some normative considerations may have beneficial effects in terms of sensitizing the community. This could be a starting point for an eclectic and multifaceted normative discussion. Moreover, such a framework is necessary to guide innovation within the field of DBS. In order to make a normative claim such as the duty or responsibility to promote technological advance, one may pursue this idea by making use of an already established ethical framework such as the one provided by Beauchamp and Childress (2013), thereby analyzing the effects of technological development on primary ethically relevant parameters. We have chosen this particular framework as a starting point for normative reflection because of its established and wide use within the clinical context. It incorporates two core bioethical principles, namely *“primum non nocere” (“First, Do No Harm”)*, reflected in the duty of nonmaleficence and beneficence, which could be described as the duty to promote the good of others. Regarding the need for and further development of neuromodulating devices, the principle of beneficence is especially ethically relevant. We believe that it is important to incorporate the values reflected in these principles into the context of industry. For one could argue that patients’ well-being may be positively affected by the implementation of technological innovation. Beneficence and nonmaleficence apply not only to the medical domain but also to that of industry and need to be balanced with the economics of the medical device industry and the need for companies to survive.

The application of these principles means that the ethical justification of any technological advance in neuromodulation depends on whether it is beneficial for patients and improves their well-being. As stated above, we argue that ethical considerations require that all stakeholders should be involved in the discussion. Accordingly, a duty to promote technological advance should not be restricted to the medical domain but also should be radially projected in order to positively affect all agents involved. If we genuinely care for the patient’s wellbeing, then the principle of beneficence should not only apply to the medical context but should have the power to transcend contextual barriers.

The goals of technological innovation and patient welfare should be complementary. Device-makers funding DBS clinical trials have an obvious interest in proving that their product can modulate neural circuits. Yet this has the potential to create a conflict of interest for researchers wanting to know how DBS affects the brain and mind of subjects if the manufacturer influences the design of the trial and the interpretation of the results. The potential for conflict is greater when the researcher has invested in such a company. Policies must be in place to prevent or at least reduce the probability of this conflict by ensuring the scientific integrity of clinical trials and that testing DBS is consistent with the interests of patients in experiencing symptom relief and improved quality of life. Ultimately, patient welfare should be the main impetus of any duty of innovation to produce more advanced neuromodulating devices (Fins and Schiff, 2010; Fins et al., 2011).

In our discussion of beneficence, we identified one pertinent principle which we thought to be easily accessible as well as used in practice. But because it is beyond the scope of this paper, we refrain from elaborating on the critical steps of balancing and weighing different values against each other. There might of course be other issues that potentially outweigh a duty of innovation under certain circumstances.

Moreover, there is a need for interdisciplinary exchange between and among representatives of patient organizations, industry, basic science, medicine and ethics in order to shape the future of DBS. A recent interdisciplinary, international forum (“brains in dialogue”, BID project)^2^ trying to foster this dialogue was unfortunately closed.

## Conclusion

DBS is an established therapeutic intervention and has recently provided hope for many patient groups including but not limited to movement disorders. Significant innovation in the field of DBS has mainly been reflected by introducing novel indications, rather than advanced technologies. We still lack a mechanistic explanation of the underlying processes involved. In turn, one has to acknowledge that the limited understanding of brain functions (bottom-up approach) poses a critical barrier to innovation. Deeper insight is crucial to further advance DBS systems towards intelligent, self-regulating closed-loop devices in combination with miniaturization and MRI-compatibility of hardware, sophisticated electrodes and programming possibilities. Furthermore, especially in the case of new indications, international joint meetings should include preclinical translational researchers as well as ethicists, recognized patient organizations, engineers and healthcare providers in neurology, neurosurgery and psychiatry for the translation of technological advances in order to create consensus of future directions of development and to improve outcomes of established indications. This consensus could be addressed to regulatory bodies for a representative impact. Finally, there is a greater need for sensitivity to the ethical duty to actively promote and demand technological advancement in neuromodulation.

## Author’s contributions

Christian Ineichen and Oguzkan Sürücü wrote the paper, Walter Glannon, Christian R. Baumann and Yasin Temel provided valuable feedback and substantially contributed to this work during the process of writing. Christian Ineichen furthermore confirms that he has final responsibility for the decision to submit for publication.

## Conflict of interest statement

The authors declare that the research was conducted in the absence of any commercial or financial relationships that could be construed as a potential conflict of interest.

## Footnotes

^1^http://www.researchandmarkets.com/reports/2538063/

^2^http://www.neuromedia.eu/NewsData.aspx?IdNews=1325&IdType=296&type=Actual
